# Tunable vector-vortex beam optical parametric oscillator

**DOI:** 10.1038/s41598-019-46016-y

**Published:** 2019-07-03

**Authors:** Varun Sharma, S. Chaitanya Kumar, A. Aadhi, H. Ye, G. K. Samanta, M. Ebrahim-Zadeh

**Affiliations:** 1 Photonic Sciences Lab., Physical Research Laboratory, Navarangpura, Ahmedabad, 380009 Gujarat India; 2 Indian Institute of Technology-Gandhinagar, Ahmedabad, 382424 Gujarat India; 3 Radiantis, Polígon Camí Ral, 08850 Gavà, Barcelona Spain; 4 ICFO-Institut de Ciencies Fotoniques, The Barcelona Institute of Science and Technology, 08860 Castelldefels, Barcelona Spain; 5 Institucio Catalana de Recerca i Estudis Avancats (ICREA), Passeig Lluis Companys 23, Barcelona, 08010 Spain

**Keywords:** Nonlinear optics, Lasers, LEDs and light sources

## Abstract

Vector-vortex beams, having both phase and polarization singularities, are of great interest for a variety of applications. Generally, such beams are produced through systematic control of phase and polarization of the laser beam, typically external to the source. However, efforts have been made to generate vector-vortex beams directly from the laser source. Given the operation of the laser at discrete wavelengths, vector-vortices are generated with limited or no wavelength tunability. Here, we report an experimental scheme for the direct generation of vector-vortex beams. Exploiting the orbital angular momentum conservation and the broad wavelength versatility of an optical parametric oscillator, we systematically control the polarization of the resonant beam using a pair of intracavity quarter-wave plates to generate coherent vector-vortex beam tunable across 964–990 nm, with output states represented on the higher-order Poincaré sphere. The generic experimental scheme paves the way for new sources of structured beams in any wavelength range across the optical spectrum and in all time-scales from continuous-wave to ultrafast regime.

## Introduction

Light beams carry angular momentum in the form of spin and orbital angular momentum (OAM). While spin angular momentum (SAM) is associated with the polarization of the light field, OAM is attested to the helicity to the Poynting vector of the optical field. Optical vortices having helical wavefront due to the azimuthally varying phase pattern represented by exp (*ilφ*) carry OAM of *lħ* per photon. Here, *φ* is the azimuthal angle and the integer, *l*, is referred as the topological charge or the order of the vortex. While optical vortices have found a great deal of interest in a variety of fields including communication^1^, lithography^2^, and high-resolution microscopy^3^, in most cases these beams are scalar vortex beams where the polarization of the beam is the same across the entire beam. However, in addition to the phase singularity, optical vortices can carry polarization singularity. Due to the vector nature (polarization), together with the vortex intensity distribution, such special class of beams are known as vector-vortex beams. Vector-vortex beams, having both phase and polarization singularities, are of great interest for a variety of applications, for example, in material processing^4^, optical trapping^5^, photon entanglement^6^, and microscopy^7^. The vector-vortices are described by points on the higher-order Poincaré sphere (HOP)^8^, representing the total angular momentum of light.

The generation of vector-vortex beams or HOP beams requires manipulation of polarization and spatial mode of a laser beam^1^. Typical techniques to generate such beams external to the optical cavity involve q-plates and phase retarders^9^, nematic spatial light modulator^10^, spatially varying retarders^11^, or interferometric methods^12^. On the other hand, efforts have been made for direct generation of such beams internal to the laser cavity by exploiting the birefringence and thermal properties of the gain medium^13^, by incorporating intracavity optical elements such as axicon^14^, or by combination of *q*-plate and quarter wave plates^15^. Unfortunately, the limited spectral coverage of lasers and material dispersion of intracavity and extracavity optical components manipulating the polarization and spatial mode of a laser beam restrict all these existing techniques, thus precluding vector-vortex beam generation over a wide spectral range.

On the other hand, optical parametric oscillators (OPOs) have been established as viable sources of tunable coherent radiation from the visible to mid-IR, covering wide spectral regions from a single device^16^. Conventionally, OPOs are exploited to generate tunable optical radiation in Gaussian spatial profile. However, in recent times, efforts have been made to generate tunable optical radiation in vortex spatial profile directly from OPOs^17,18^ by harnessing the OAM conservation in the parametric process. Similarly, multimode OPOs have been demonstrated as suitable tools to generate hyper-entangled states^19–21^. Moreover, one can actively control the generation of such scalar optical vortices in the signal and/or idler depending upon the loss parameter^17,22^ in OPO systems and also controlling the symmetry of OAM in a driven OPO^23^. On the other hand, efforts have been made to generate OAM at different wavelengths through various nonlinear processes^24–28^ including second-harmonic-generation (SHG), sum-frequency-generation, high-harmonic-generation, and optical parametric generation using both birefringence-phase-matching and quasi-phase-matching techniques and control the OAM in SHG process by selective choice of the input polarization of the pump beam^29^. Using OPOs providing scalar vortex beams, one can, in principle, manipulate the polarization of the intracavity field to generate vector-vortex beams directly from OPOs. However, the phase-matching condition is critically dependent the polarization of the interacting beams, dictating the parametric gain for the nonlinear process. Therefore, manipulating the polarization of intracavity beams in an OPO, as required for vector-vortex generation, is very challenging.

**Figure 1:**
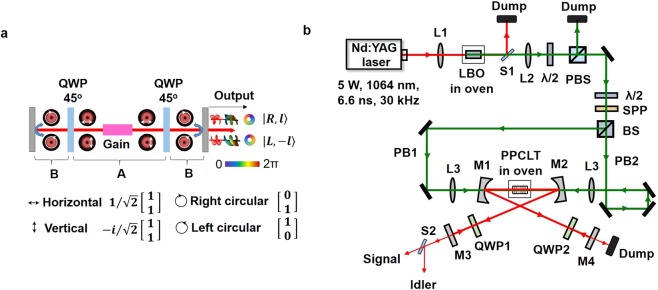
Vector- vortex beam optical parametric oscillator. (**a**) Pictorial representation of the evolution of polarization and OAM states of the resonant beam, through one roundtrip in the OPO cavity, horizontal, vertical, right circular and left circular represent the polarization of resonating beam light. (**b**) Schematic of the experimental setup. *λ*/2, half-wave plate; PBS, polarizing beam-splitter; BS, 50:50 beam-splitter; SPP, spiral phase plate; L1-3, lenses; M1-4, OPO mirrors; QWP1-2, quarter-wave plates; LBO, PPCLT, nonlinear crystals; S1-2, wavelength separators.

In this report, we demonstrate a generic experimental scheme to generate tunable vector-vortex beam directly from an OPO. Taking advantage of the directional property of the OPO (nonlinear gain in the direction of the pump beam), we manipulate the resonating beam in such a way that the polarization state is preserved after every round-trip, enabling efficient frequency conversion in the presence of the pump beam. Consider a standing-wave Fabry-Perot cavity, as shown in Fig. 1(a). The nonlinear gain can be achieved in both clockwise and counter-clockwise directions with the resonating beams having vortex spatial structures when the gain medium is pumped with vortex beam of linear polarization (say, vertical) from both sides. The inclusion of two quarter-wave plates (QWP) with optic axis rotated by 45° with respect to horizontal axis ensures that the polarization state is always linear in region A and circular in region B. In every round-trip, the resonating beam in region A returns to its initial polarization state (vertical) to ensure efficient frequency conversion in the gain medium. Here, the manipulation of polarization and spatial mode of the output beam, as required for vector-vortex beams, is achieved by the intracavity QWP and the OAM conservation in the parametric process, respectively. Using such a scheme in a nanosecond OPO in doubly-resonant oscillator (DRO) configuration and pumped in both directions by optical vortex beam, we generate vector-vortex beam of order, *l* = 1, tunable over 964–990 nm with an output power in excess of 5 mW.

## Experiment

The schematic experimental setup is shown in Fig. 1(b). A Q-switched Nd:YAG laser producing 5 W of average power at 1064 nm in 6.6 ns pulses at a repetition rate of 30 kHz, as also used in ref.^30^, works as the fundamental source. Using a lens (L1), the fundamental laser is frequency-doubled in a 30-mm-long LBO crystal, providing up to 1.5 W of green power at 532 nm in Gaussian intensity profile. After separation from the fundamental using a dichroic mirror (S1), the green pump beam is collimated using a lens (L2), with the input power to the experiment controlled using a combination of a half-wave plate (*λ*/2) and a polarizing beam-splitter (PBS) cube. A spiral phase plate (SPP) is used to convert the green Gaussian pump beam into an optical vortex of order, *l*_*p*_ = ±1. The green pump vortex is divided into two beams, PB1 and PB2, of equal intensity, by using a non-polarizing 50:50 beam-splitter (BS) cube. Further, the two beams are steered to the DRO with equal number (three) of reflections, in order to maintain the same vortex polarity, *l*_*p*_ = 1, for both PB1 and PB2. The path length of the pump beams is also maintained to be identical. The DRO is configured in a four-mirror standing-wave cavity with two concave mirrors, M1-M2 (*r* = 100 mm), and two plane mirrors, M3 and M4, with a total cavity length of 33.5 cm, allowing six roundtrips of the resonant waves over the 6.6 ns pulse duration. All mirrors are antireflection-coated for high transmission (*R *< 5%) at 532 nm and high reflectivity (*R* > 95%) across 950–1200 nm, ensuring DRO operation over the signal and idler wavelength range^17^. A 30-mm-long, 1-mm-wide, and 0.5-mm-thick periodically-poled congruent LiTaO_3_ (PPCLT) crystal with a single grating period of *Λ*_*c*_ = 7.91 μm, placed between M1 and M2, is used as the nonlinear crystal. The crystal is housed in an oven, adjustable from room temperature to 200 °C with a stability of ±0.1 °C. The *λ*/2 plate before SPP adjusts the pump beam polarization for optimum phase-matching of PPCLT crystal. The nonlinear crystal is pumped from both sides using two identical lenses (L3) of focal length, *f* = 150 mm, ensuring identical mode-matching and all cavity parameters for OPO operation in both clockwise and counter-clockwise directions. QWP1 and QWP2, optimized for the signal wavelength centered at 980 nm, placed inside the DRO cavity, are used to manipulate the polarization state of the resonating beam. The leakage signal and idler of M3 are separated by the separator, S2, and recoded by CCD and pyro-camera, respectively.

The working principle of our experimental scheme for the vector-vortex beam DRO as also presented in ref.^30^, can be understood as follows. The PPCLT crystal is designed for type-0 (*e* → *e* + *e*) quasi-phase-matched interaction with vertical polarization states for the pump, signal and idler. On the other hand, the OAM of the pump beam with vortex order, *l*, can be transferred either to the signal or idler through systematic control of the cavity losses^17^. Therefore, due to higher losses of the cavity mirrors at the idler wavelength than the signal, the pump OAM is transferred to the signal, while idler carries order, *l* = 0 (Gaussian). Under this condition, the pump beam, PB1, creates signal with the state (polarization and OAM) of |V, *l* 〉, and the corresponding idler state of |V, 0〉. As each reflection flips the OAM from +*l* to −*l* and vice-versa, the states of the signal beam before and after the QWP1, with optic axis at +45°, are |V, −*l*〉 and |R, −*l* 〉, respectively. The mirror, M3, on reflection, changes the state of the signal beam into |L, *l*〉, which, on passing through QWP1, is converted into |H, *l* 〉. Here, V and H correspond to the vertical and horizontal polarization, while R and L represent the right and left circular polarizations. Similarly, the state of the signal beam before QWP2, and after successive reflection by M2 and M1 is |H, *l* 〉, which on propagation through QWP2, reflection by M4, and retrun-pass through QWP2 transformed into |L, l >, |R,-l >, and |V,-l >, respectively. As a result, the signal beam produced by the pump, PB1, after one round-trip returns to its original state, |V, *l* 〉, and is amplified by the pump beam. Similarly, the signal beam generated by the pump beam, PB2, of state |V, *l* 〉, has the state of |L, *l* 〉 and |R, −*l* 〉 before M3 and M4, respectively. As a result, the signal beam out-coupled from M3 and M4 has state, |R, −*l*〉 + |L, *l*〉, which is the final state of the generated vector-vortex beam. Since QWP1 and QWP2 are not optimized at the idler wavelengths, in each round-trip the idler beam of vortex order, *l* = 0, generated by both pump beams, will encounter extra losses due to the improper (elliptical) polarization rotation by the QWPs, which in turn results in the idler carrying OAM of order, *l* = 0, satisfying the OAM conservation in DRO^17^.

## Results and Discussions

**Figure 2:**
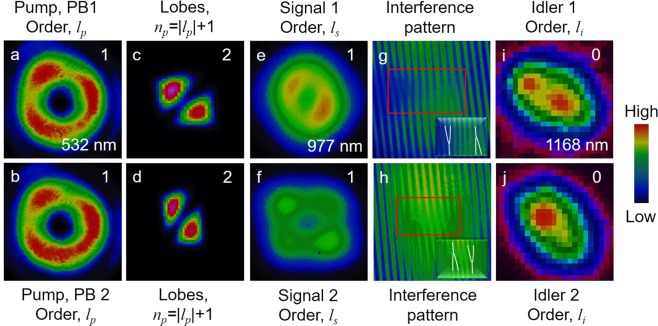
Generation of vortex beam. (**a**,**b**) Intensity profiles and (**c**,**d**) lobe structure of the pump beams, PB1 and PB2. (**e**,**f**) Intensity distribution and corresponding (**g**,**h**) self-interference fringe pattern of the signal beams generated by PB1 and PB2. (**i**,**j**) Idler intensity profiles generated by PB1 and PB2.

**Figure 3:**
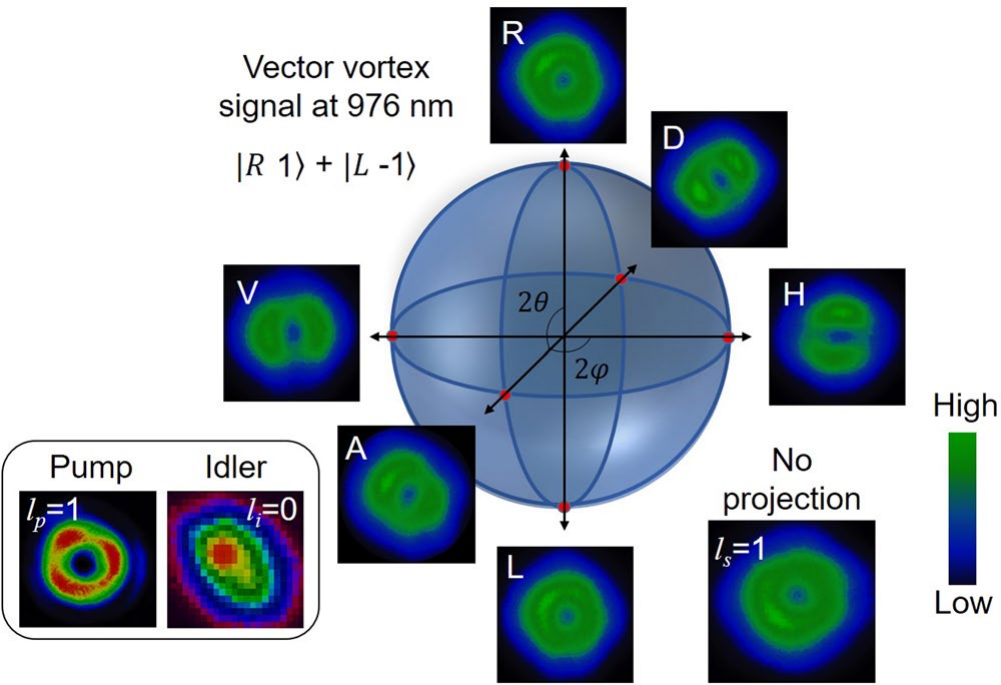
Intensity profiles of the signal vector-vortex beam of OAM mode, *l*_*s*_ = 1. Depending on the projection of the beam in different polarization states, R, A, L, D, H and V (as shown by the white letters on the images) on the Poincaré sphere, the mode pattern of the beam recorded by the CCD camera change at different points on higher-order Poincaré (LG-Bloch) sphere for 1/√2 (|R, 1〉 + *e*^*−iφ*^ |L, −1〉). Inset images are intensity distribution of one of the pumps, idler and the signal beams.

To study the OAM exchange mechanism of the nanosecond DRO, we fixed the crystal temperature at 77 °C, corresponding to the signal (idler) wavelength of 977 nm (1168 nm). Pumping the crystal with PB1 and PB2, separately, with an average power of 300 mW and vortex order of *l*_*p*_ = 1, we recorded the spatial intensity profile of the signal and idler beams, with the results shown in Fig. 2. As expected, both the pump beams, PB1 and PB2, derived from a single pump beam, carry the same doughnut-shaped intensity profile (first column, (a, b), of Fig. 2). The number of characteristic lobes, (*n*_*p*_ = |*l*_*p*_| +1), and their orientation, as shown in second column, (c, d), of Fig. 2, recorded using the tilted lens technique^17^, confirm that both the pump beams (PB1 and PB2) carry vortex beam of same order, |*l*_*p*_| = 1, and, same helicity (sign). The third column, (e, f), of Fig. 2, shows the intensity profile of the signal beams generated by PB1 and PB2, respectively. The signal beams have doughnut-shaped intensity profile with central minimum, resembling a vortex beam, which is further confirmed by the characteristic fork pattern of the vortex-vortex interference recorded using Mach-Zehnder interferometer. As evident from the fork pattern and their orientation, shown in fourth column, (g, h), of Fig. 2, both the signal beams carry vortex of order, |*l*_*s*_| = 1, but with opposite sign (±). The opposite sign of the signal vortices, as required for vector-vortex generation, can be attributed to the odd and even number of reflections experienced by the signal beams generated by PB1 and PB2 vortices of same sign, respectively, before transmission through the mirror, M3. Unlike the pump vortices, the signal vortices have background intensity at their dark core, which is due to the presence of the OPG signal generated by the high gain of the PPCLT crystal under nanosecond laser pumping. In our experimental setup, we observed OPG of the vortex pump beam at an input power of 350 mW. For OPO operation, we adjusted the pump power below the OPG threshold. Under this condition, we still observed background OPG signal due to the high intracavity power. The fifth column, (i-j), of Fig. 2, shows the intensity profiles of the corresponding idler beams in Gaussian intensity profile, without any dark core in the beams. The elliptic beam profile of the idler is attributed to tilts in the collection optics. Such observation clearly confirms that in nanosecond vortex-pumped DRO, the pump OAM is directly transferred to the signal beam (*l*_*s*_ = *l*_*p*_), while the idler beam maintains Gaussian spatial mode (*l*_*i*_ = 0), owing to OAM conservation (*l*_*p*_ = *l*_*s*_ + *l*_*i*_) in the parametric process^17^. The pixelated image of the idler intensity distribution can be attributed to the large pixel size of the pyro-camera. Knowing the generation of signal scalar-vortex beams for both pump beams (PB1 and PB2), using a spectrometer, we measured the wavelength of signal beams to be 977 nm separately for both pumps. However, with both pump beams present in the crystal, the resultant signal beam spectrum shifted by 1 nm to 976 nm. Such shift in the signal spectrum could be attributed to a minor temperature change (~0.6 °C) in the crystal due to high intracavity signal as well as the pump power. Since both signal beams have same wavelength and polarization, we expect coherent coupling between the signal pulses generated by both pump beams^31^. The threshold of the DRO for Gaussian pumping in the absence of SPP was measured to be ~75 mW for each pump beam in either direction. However, in presence of both pump beams, the DRO threshold decreased to ~40 mW, clearly confirming coherent coupling between the generated signal pulses. It also confirms the temporal and spatial overlap of the resonating signal beams of both pumps, as required for vector-vortex generation. As expected^32^, the DRO threshold for vortex pump beams, PB1 and PB2, separately, was measured to be ~290 mW, higher than that for the Gaussian-beam-pumped DRO. While a similar threshold reduction due to coherent coupling is also observed for vortex pumping, the signal beam loses its vorticity as the signal pulses generated from PB1 and PB2 are of opposite helicities. However, for vector-vortex generation we inserted QWPs inside the cavity, while pumping the DRO from both sides with an average power of 300 mW in each beam. Both QWPs (QWP1 and QWP2) had optic axis rotated by +45° with respect to horizontal axis. We analyzed the out-coupled signal from mirror, M3, using a detection unit comprising the combination of QWP, *λ*/2 plate, and a PBS^6^. We projected the signal vector-vortex beam of OAM mode, *l* = 1, in different polarization states, horizontal (H), vertical (V), diagonal (D), anti-diagonal (A), right circular (R), and left circular (L), on the Poincaré sphere, by adjusting the optic axis of the QWP and *λ*/2 plate. The results are shown in Fig. 3. As evident from Fig. 3, the projection of signal beam to R and L polarization states results in vortex intensity profile with OAM order, *l* = 1 and *l* = −1, respectively. However, the projection on H, V, A and D polarization states result in the superposition of two opposite helical wave fronts of vortex order, |*l*| = 1, generating a ring lattice structure containing two-lobe (2 *l*) pattern at different orientations. All these projected intensity distributions represent different points on the Poincaré sphere. The rotation of the two-lobe pattern obtained with the rotation of the optic axis of the QWP and *λ*/2 plate for polarization measurement determines the spatial distribution pattern of the vector-vortex beam and verifies the non-separability. The inset of Fig. 3 shows the intensity profiles of the pump and idler beams without any polarization projection. As in previous reports^15,33^, one can measure the purity of the OAM mode of the vector- vortex beam through the mode projection technique^1,34,35^. However, due to a lack of suitable spatial light modulator (SLM) in our laboratory, we were not able to perform the mode purity measurement for the vector- vortex beams. On the other hand, since the OAM beam of the OPO is generated through the OAM conservation in nonlinear process, we expect the generated beam to carry the pure OAM mode of the pump. Additionally, the polarization projection and beam quality measurements are performed in the far field region to reduce the effect of higher- order cavity modes (if any) in the resonant beams.

**Figure 4:**
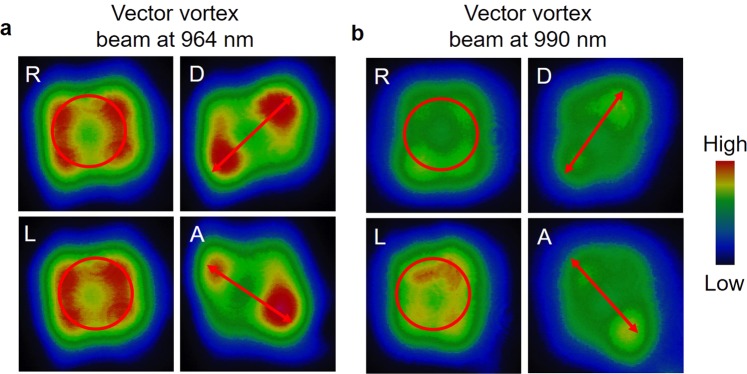
Vector-vortex beam at two different wavelengths, 964 and 990 nm, across the tuning range of the OPO. Intensity distribution of the vector-vortex beams for the projective measurement in polarization states, R, L, D, and A for the vector-vortex beams at (**a**) 964 nm and (**b**) 990 nm. The projective measurement in A and D basis results 2*l* petals.

Confirming the generation of vector-vortex beam from the OPO at 977 nm, we further studied the performance of the device across the tuning range. Pumping the OPO from both sides, we changed the crystal temperature to vary the signal and idler wavelengths. Although the current OPO can be tuned across 850–1420 nm^31^, the cavity mirrors limit the operation to 950–1200 nm. On the other hand, the retardance of the intracavity QWPs is the limiting factor for the wavelength tunability of the vector-vortex output. Since the zeroth-order QWPs used in the current study have retardance varying from 0.2523 (0.20) to 0.2447 (0.208) with the signal (idler) wavelength variation from 960 nm (1193 nm) to 990 nm (1149 nm), the tuning is confined to this wavelength range. Such small change of retardance with wavelength maintains linear polarization of the resonating signal beam in region A (see Fig. 1a) of the OPO without introducing significant losses due to polarization rotation. However, the inappropriate retardance of the QWP changes the linear polarization into elliptic, severely effecting the phase-matching condition. One can, in principle, use achromatic QWPs having constant retardance over a wider range of wavelengths to increase the tunability of the vector- vortex beam. However, we did not have available such achromatic QWP to extend the wavelength range of the current vector -vortex OPO. We studied the non-separability of the signal beam at two different wavelengths, 964 nm and 990 nm, across the tuning range. To confirm the vector-vortex nature of the signal pulses in this range, we again performed projective measurements by using a QWP and PBS, and projected the generated state in different polarization basis, with the results shown in Fig. 4. As evident from Fig. 4(a,b), the rotation of the QWP for polarization measurement rotates the spatial intensity distribution of signal beams at 964 nm and 990 nm, thus verifying the non-separability and vector-vortex nature of the signal beam across the tuning range of 964–990 nm. Due to the high reflectivity of the cavity mirrors, we measured an out-coupled vector-vortex beam power of >5 mW in 4.4 ns pulses of 0.25 µJ energy across the tuning range. However, with optimization of output coupling, a substantial enhancement in vector-vortex beam power are expected, enabling practical applications certainly at the cost of increase of operation threshold of the OPO.

## Conclusions

In conclusion, we have demonstrated an experimental scheme to generate vector-vortex beam directly from an OPO in DRO configuration, pumped by vortex beams from both sides of the cavity. By manipulating the polarization of the resonant wave using a pair of intracavity QPWs, we have generated vector-vortex beam tunable across 964–990 nm. Wavelength tunability of the source is limited by the uniform retardance range of the QWPs. Further increase in the tunability of the vector-vortex beam can be achieved by using broadband achromatic zero-order QWP. The generic approach can also be used in cw and ultrafast OPOs and extended to other wavelength regions across the optical spectrum.
